# A Case of Unilateral Partial Testicular Rupture Misdiagnosed as Epididymo-Orchitis

**DOI:** 10.7759/cureus.92387

**Published:** 2025-09-15

**Authors:** Sandeep Kumar, Arun JP George, Nirmal Thampi John

**Affiliations:** 1 Urology, Christian Medical College Vellore, Vellore, IND

**Keywords:** emergency surgical care, epididymo-orchitis, general trauma surgery, testicular rupture, urological trauma

## Abstract

Testicular rupture is a rare but serious complication of blunt scrotal trauma that necessitates urgent intervention. A clinical examination of the scrotum alone cannot facilitate the diagnosis. Misdiagnosis can lead to delayed treatment and poor testicular salvage. Here, we report a case of a partial left testicular rupture in a patient initially managed as epididymo-orchitis, who subsequently underwent successful scrotal exploration and tunica albuginea repair. This case report re-emphasizes the importance of a thorough history, clinical assessment, and ultrasound imaging in the early detection and successful management of the condition.

## Introduction

Blunt trauma to the scrotum can result in testicular rupture, a urological emergency characterized by disruption of the tunica albuginea and extrusion of testicular contents [1]. The severity of blunt scrotal trauma ranges from tears of tunica to complete organ loss [2]. Although not lethal, the loss of a testicle can compromise fertility and negatively influence social behavior, especially among teenagers [3]. Testicular rupture refers to a tear in the fibrous sheath of the testis, known as the tunica albuginea, resulting in the extrusion of seminiferous tubules into the scrotal sac [4]. Patients typically present with nonspecific symptoms, such as acute scrotal pain and swelling, nausea, and sometimes vomiting [3]. The condition may be underdiagnosed, especially when pain and swelling are attributed to more common conditions like epididymo-orchitis. Prompt diagnosis using ultrasonography and early surgical intervention are key to testicular preservation [5]. Treatment generally involves surgical exploration with debridement or orchidectomy [5,6]. However, in selected cases, conservative management has gained recognition as a viable alternative in recent years [5,6]. This report underscores the diagnostic value of ultrasound and reinforces the importance of surgical exploration in cases of acute scrotum with partial testicular rupture.

## Case presentation

A 20-year-old male sustained blunt trauma to the left hemiscrotum following impact with a bicycle handle. In the immediate aftermath, he experienced severe pain and progressive swelling of the left scrotum, but had no associated urinary complaints. He sought care at a local healthcare facility, where he was managed conservatively with analgesics and scrotal support under the clinical impression of epididymo-orchitis, based on history and left hemiscrotal swelling. Four days later, he presented to our institution with increased scrotal swelling, though the pain had subsided, and he was afebrile. He had no significant past medical history or associated comorbid illnesses.

On clinical examination, the patient was hemodynamically stable with a pulse rate of 90 beats per minute and a blood pressure of 120/80 mmHg. General and systemic examinations were unremarkable. Abdominal examination revealed a soft, nontender abdomen with no evidence of organomegaly, mass, or hernia. On local examination, the external genitalia were normal. The left testis was in a normal position but appeared enlarged, measuring approximately 8×5 cm (normal: 4×3×2 cm). It was firm in consistency, nontender, and showed no signs of erythema or local rise in temperature. The right testis was normal in size, position, and consistency, and was nontender without any inflammatory signs. Bilateral cremasteric reflex was normal.

**Figure 1:**
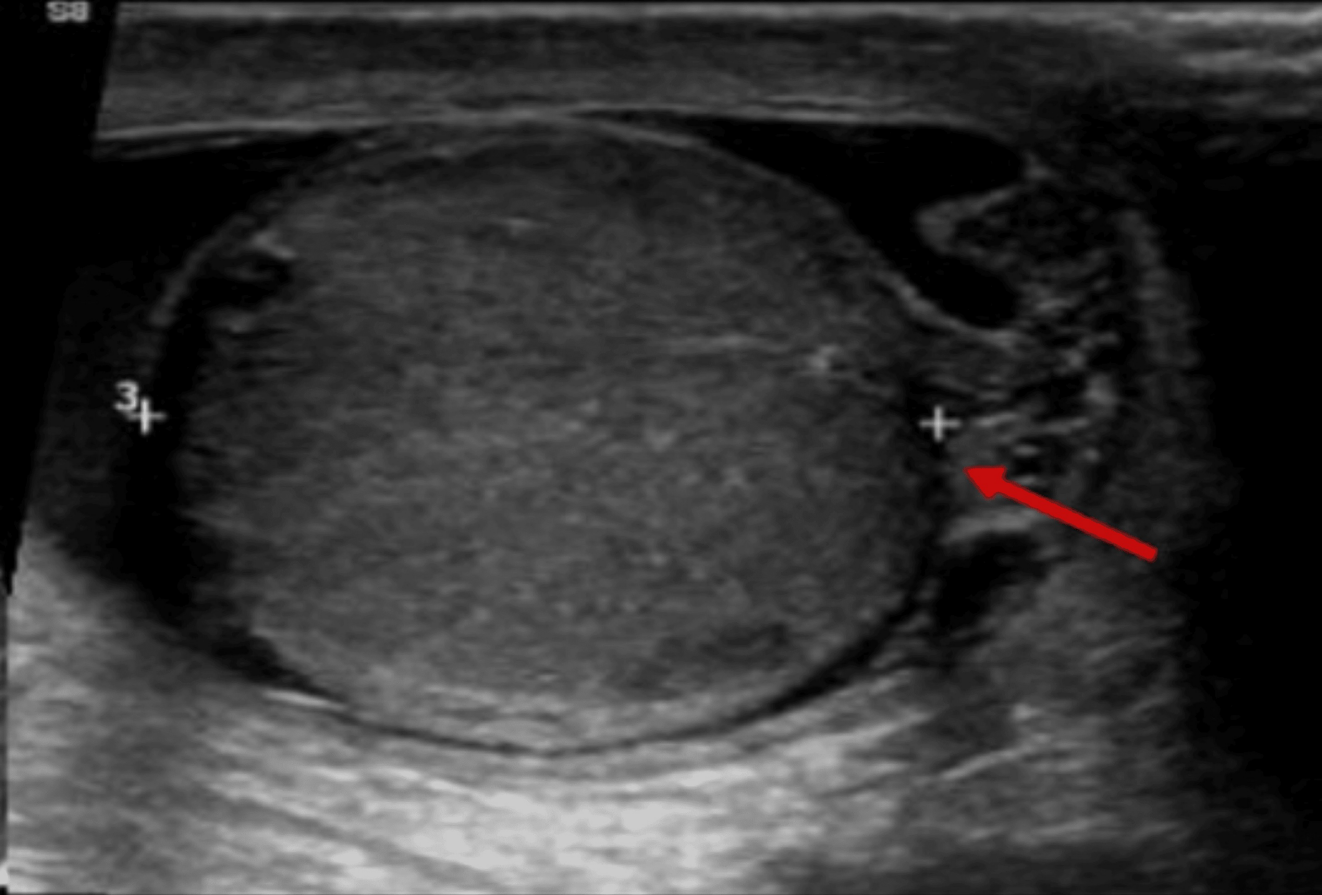
Ultrasound of scrotum showing discontinuity of tunica albuginea (arrow) at the inferior pole of the left testis.

Laboratory investigations, including urinalysis, complete blood count, and total leukocyte count, were within normal limits. Scrotal ultrasonography revealed a partial rupture of the left testis with an associated hematocele, while testicular vascularity was preserved (Figure 1).

**Figure 2:**
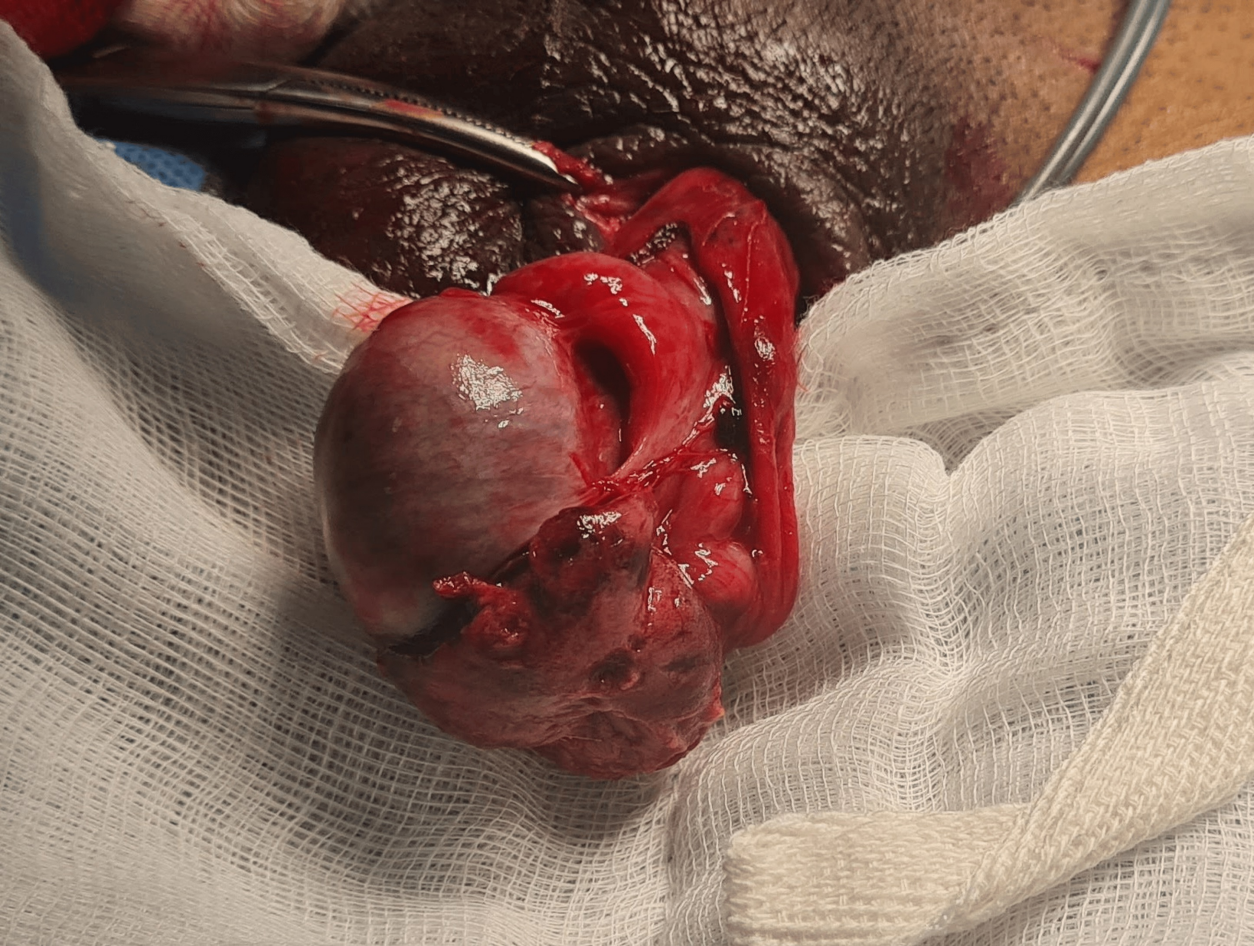
Intraoperative image showing inferior pole tunica albuginea rupture with necrotic left testicular tissue.

Testicular torsion is an important differential diagnosis in the present scenario. The patient presented to us four days following the trauma, and so screening with an ultrasound was justified. Doppler studies revealed normal flow patterns, thus excluding the possibility of torsion. The patient was taken up for left scrotal exploration under regional anesthesia. Upon opening the tunica vaginalis, approximately 10-15 mL of hematocele was evacuated. Intraoperative findings revealed a tear in the inferior aspect of the tunica albuginea, with extrusion of devitalized testicular parenchyma (Figure 2).

**Figure 3:**
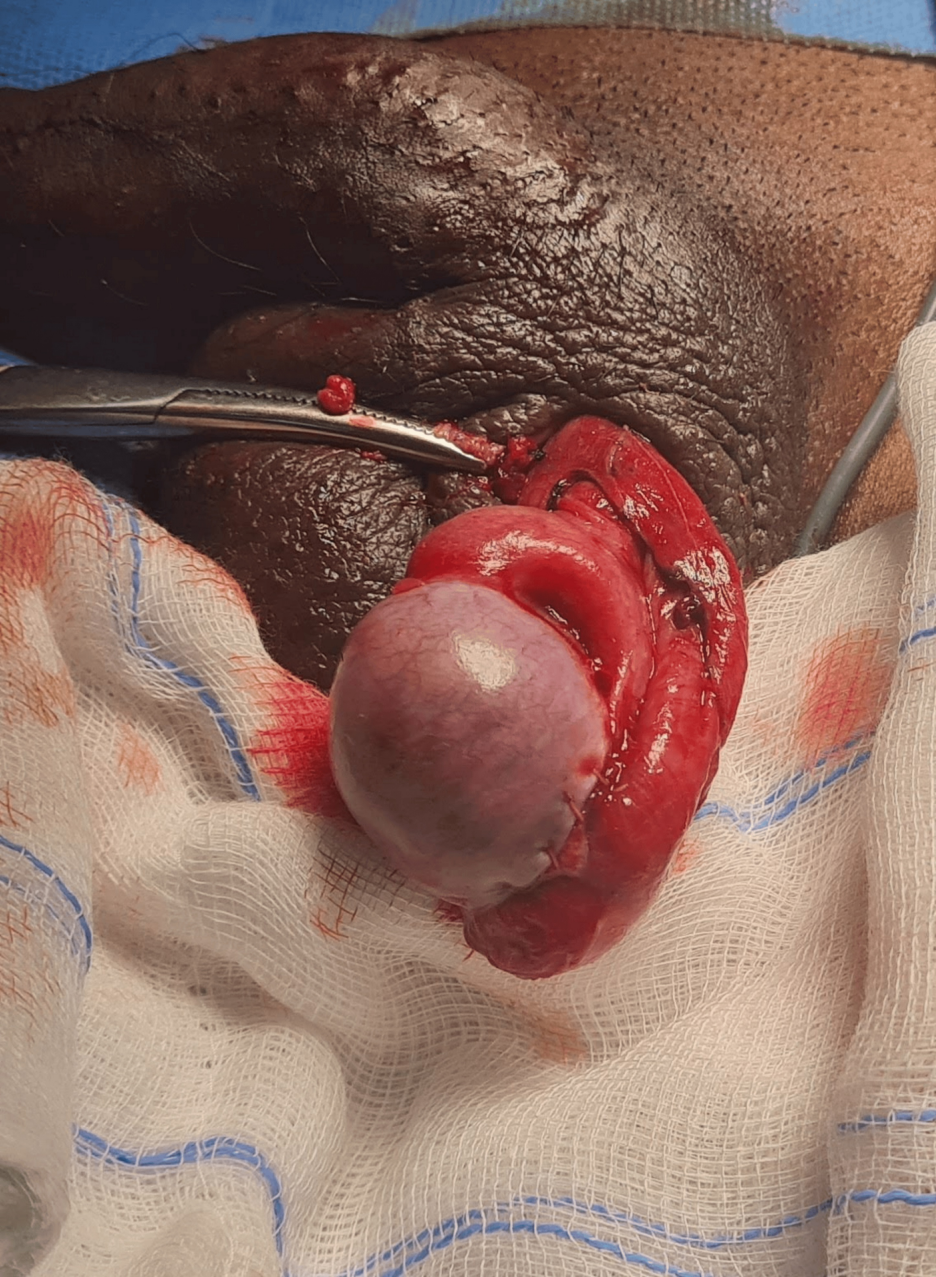
Intraoperative image after repair of the tunica albuginea of the left testis.

The remaining testicular tissue appeared viable, with preserved vascularity. The necrotic tissue was carefully debrided, and primary repair of the tunica albuginea was performed using absorbable polyglactin sutures (Figure 3).

**Figure 4:**
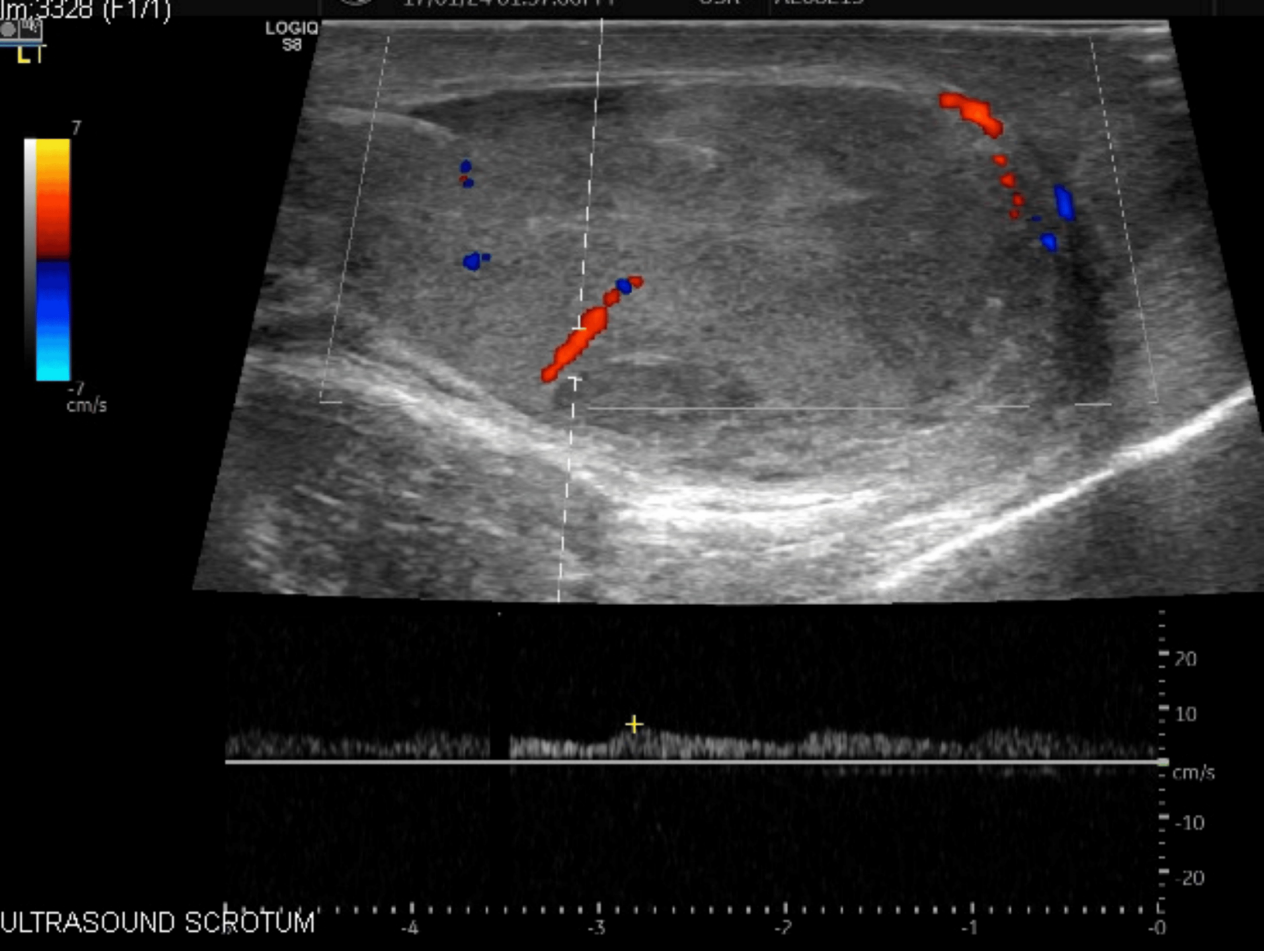
Postoperative ultrasound image showing the left testis with preserved vascularity.

Layered closure of the dartos and scrotal skin was subsequently completed. Postoperative recovery was uneventful. A follow-up ultrasound at one month revealed a normal left testis with preserved vascularity and negligible residual hematoma or fluid collection (Figure 4).

## Discussion

Testicular rupture occurs in approximately 50% of blunt testicular trauma cases requiring surgical exploration [1]. Although injuries to the scrotum are not frequently encountered in emergency practice, they remain a notable cause of acute scrotal pain [5].

Such trauma is most often sustained by young adult males during sports, straddle injuries, or motor vehicle accidents [6,7]. Blunt force can result in hematoma formation, testicular rupture, or intratesticular fractures [8,9]. Excessive intratesticular pressure created by blunt force may exceed the strength of the tunica albuginea, leading to its disruption and extrusion of testicular tissue [5].

Clinical examination may elicit tenderness, scrotal swelling, and ecchymosis with a normal cremasteric reflex [10]. A high index of suspicion is essential, particularly when a blunt trauma history is present. Early scrotal ultrasonography has high sensitivity and specificity in diagnosing rupture [5]. Ultrasound scrotum gives the contour discontinuities of the testis and distortion of parenchymal echotexture [7]. Color Doppler ultrasonography is especially valuable, as preserved vascularity indicates viability and aids in guiding treatment decisions. When contour irregularity is combined with heterogeneous parenchyma, the sensitivity and specificity for diagnosing testicular rupture may reach up to 100% and 93.5%, respectively [7].

In this case, delayed presentation and misdiagnosis led to four days of conservative management, during which testicular tissue necrosis likely progressed. Our patient presented with a partial tunica albuginea tear with hematocele following blunt trauma, confirmed on imaging and intraoperatively. Fortunately, viable tissue remained, allowing successful repair and preservation of the testis. Timely surgical intervention improves salvage rates, reduces the risk of infection, and preserves endocrine and reproductive function [11]. However, delayed intervention is associated with higher orchidectomy rates, approaching 45% [1,12].

There is still debate on whether immediate surgery is always mandatory. Some studies caution that aggressive debridement may remove viable tissue, and rapid closure of the tunica albuginea could lead to testicular atrophy [12]. Others emphasize that patients treated surgically may still experience pain or atrophy [13]. Nevertheless, most reports favor exploration within 72 h to maximize salvage, with testicular preservation rates dropping from 80-90% to 45-55% if delayed beyond this period [5,14,15]. In our patient, although the presentation was delayed beyond 72 h due to an initial misdiagnosis, surgical exploration remained crucial, allowing for the successful salvage of the testis despite the late intervention.

## Conclusions

This case highlights the critical importance of maintaining a high index of suspicion for testicular rupture in patients presenting with scrotal pain. Early scrotal ultrasonography remains a valuable diagnostic tool, enabling a prompt diagnosis. Delayed diagnosis risks progressive necrosis and potential testicular loss. Primary repair of the tunica albuginea preserves testicular endocrine and reproductive function. This case reinforces that a thorough trauma history, rapid imaging, and early surgical intervention are critical to optimize outcomes in blunt scrotal injuries, reduce morbidity, and ensure the highest possible testicular salvage rates.
